# Neural changes associated with cerebellar tDCS studied using MR spectroscopy

**DOI:** 10.1007/s00221-018-5170-1

**Published:** 2018-02-05

**Authors:** Roya Jalali, Alimul Chowdhury, Martin Wilson, R. Chris Miall, Joseph M. Galea

**Affiliations:** 1 Physical Sciences of Imaging in the Biomedical Sciences (PSIBS), University of Birmingham, Birmingham, B15 2TT UK; 2 School of Psychology, University of Birmingham, Birmingham, B15 2TT UK; 3 Medical Physics, University Hospitals Birmingham NHS Foundation Trust, Birmingham, B15 2TH UK

**Keywords:** Cerebellum, GABA, Glutamate, MR spectroscopy, Motor adaptation, Non-invasive stimulation

## Abstract

Anodal cerebellar transcranial direct current stimulation (tDCS) is known to enhance motor learning, and therefore, has been suggested to hold promise as a therapeutic intervention. However, the neural mechanisms underpinning the effects of cerebellar tDCS are currently unknown. We investigated the neural changes associated with cerebellar tDCS using magnetic resonance spectroscopy (MRS). 34 healthy participants were divided into two groups which received either concurrent anodal or sham cerebellar tDCS during a visuomotor adaptation task. The anodal group underwent an additional session involving MRS in which the main inhibitory and excitatory neurotransmitters: GABA and glutamate (Glu) were measured pre-, during, and post anodal cerebellar tDCS, but without the behavioural task. We found no significant group-level changes in GABA or glutamate during- or post-tDCS compared to pre-tDCS levels, however, there was large degree of variability across participants. Although cerebellar tDCS did not affect visuomotor adaptation, surprisingly cerebellar tDCS increased motor memory retention with this being strongly correlated with a decrease in cerebellar glutamate levels during tDCS across participants. This work provides novel insights regarding the neural mechanisms which may underlie cerebellar tDCS, but also reveals limitations in the ability to produce robust effects across participants and between studies.

## Introduction

Numerous studies have shown a facilitatory effect of anodal cerebellar transcranial direct current stimulation (tDCS) on both motor and cognitive behavioural tasks (Galea et al. 2009; Grimaldi et al. 2014; Cantarero et al. 2015). For instance, Galea et al. (2011) applied anodal cerebellar tDCS during visuomotor adaptation and found anodal cerebellar tDCS led to faster adaptation, relative to either primary motor cortex (M1) anodal tDCS or sham tDCS (Galea et al. 2011). This effect on motor adaptation/learning has been replicated in visuomotor adaptation (Hardwick and Celnik 2014; Block and Celnik 2013; Doppelmayr et al. 2016; Leow et al. 2017), force-field adaptation (Herzfeld et al. 2014), locomotor adaptation (Jayaram et al. 2012), saccade adaptation (Panouilleres et al. 2015; Avila et al. 2015), motor skill learning (Cantarero et al. 2015), and language prediction tasks (Miall et al. 2016). As a result, it has been suggested that cerebellar tDCS is not only a useful tool to understand cerebellar function but also as a possible clinical technique to restore cerebellar function in patients suffering from cerebellar-based disorders (Grimaldi et al. 2014). However, there are also inconsistencies regarding the impact of cerebellar tDCS with several studies reporting cerebellar tDCS to have little or no effect on motor learning (Conley et al. 2016; Minarik et al. 2016; Jalali et al. 2017) or large variability between- and within-subjects (Dyke et al. 2016). Therefore, understanding the underlying causes of this variability is essential.

Previous work has investigated the neural changes associated with M1 anodal tDCS using a range of MRI techniques (Stagg et al. 2011; Kim et al. 2014; Antal et al. 2011; Hunter et al. 2015; Kunze et al. 2016). For example, magnetic resonance spectroscopy (MRS) revealed that M1 anodal tDCS caused a decrease in gamma-aminobutyric acid (GABA), with the magnitude of this decrease being correlated with improvements in both sequence learning (Stagg et al. 2011) and force-field adaptation (Kim et al. 2014), but they did not report any significant change in Glu or any correlation between the change in Glu and motor behaviour.

Despite this work relating to M1 tDCS, no previous research has attempted to use MRS to investigate the neural changes observed with cerebellar tDCS. Given the abundance of GABA and Glu within the cerebellar cortex (Waddell et al. 2011), we predicted that these were the two metabolites most likely to be affected by anodal cerebellar tDCS. Therefore, using MRS, the changes in GABA and Glu were quantified within the right cerebellar cortex directly underneath the anodal electrode pre, during and post tDCS. We sought to understand if there is any detectable change in GABA or Glu in response to cerebellar tDCS and if their alteration could predict individual differences in the effect of cerebellar tDCS on visuomotor adaptation performance. According to previous findings, we hypothesised that a reduction in GABA induced by cerebellar anodal tDCS would be positively correlated with the degree of visuomotor adaptation.

## Materials and methods

### Participants

34 healthy young individuals participated in this study (mean age: 22 ± 2 years; 11 male) and were divided into two groups of 17: anodal (23 ± 5 years; 8 male) and sham (19 ± 2 years; 3 male). All were naïve to the behavioural task, self-assessed right handed, had normal/corrected vision, and reported to have no history of any neurological condition. The study was approved by the Ethical Review Committee at the University of Birmingham and was in accordance with the declaration of Helsinki. Written informed consent was obtained from all participants after screening for suitability for MR imaging and brain stimulation. Participants were recruited through online advertising and received monetary compensation.

All participants first completed a behavioural task, testing visuomotor adaptation during active or sham TDCS. At the end of the behavioural session, 29 of the 34 participants reported their attention, fatigue, and quality of sleep using a questionnaire with a scale from 1 to 7. They also reported whether they believed they had received active or sham stimulation, and their hours of sleep during the previous night (Table 1). After completing the behavioural task, all 17 participants from the anodal group underwent a session of MRS, with concurrent tDCS. The sham group were not imaged.

### Transcranial direct current stimulation (tDCS)

For the behavioural session, anodal tDCS (DC-Stimulator, NeuroConn, Germany) was delivered through a pair of rubber electrodes (4 × 4 cm^2^) within two 5 × 5 cm^2^ pads soaked in a saline solution (Wagner et al. 2014) and attached to the head with Coban self-adhesive tape. The anodal electrode was placed over the right cerebellar cortex, 3 cm lateral to the inion. The cathodal electrode (reference) was placed over the right buccinator muscle (Galea et al. 2011) as it has been shown to be an effective montage for cerebellar stimulation (Rampersad et al. 2014). At the onset of stimulation, current was increased in a ramp-like fashion over a period of 10 s. For the behavioural study, in the anodal group, a 2 mA current (current density *J* = 0.08 mA/cm^2^) was applied for 25 min. In the sham group, tDCS was ramped up over period of 10 s, remained on for 10 s before being ramped down and switching off. Participants during the behavioural task were blinded to whether anodal or sham was applied (Table 1).

For the MR session, 1.8 mA anodal tDCS was delivered (*J* = 0.07 mA/cm^2^) through a pair of rubber electrodes (5 × 5 cm^2^). The electrodes were attached to each participant’s head, in the same position as the behavioural session, using EEG paste and Coban self-adhesive tape. Electrodes were connected to an MR-compatible tDCS machine (DC-Stimulator-MR, NeuroConn, Germany). Ideally 2 mA stimulation would have been used, however, high impedance (> 55 kΩ) within the MRI-compatible tDCS equipment meant this was not possible.

To avoid MR image artefacts, the tDCS current was set at 0 mA for the pre-and post-stimulation data acquisition, rather than switching the tDCS device off. This was because the tDCS device employed two filters to prevent leakage of radio-frequency electromagnetic fields into the MRI faraday cage, which operated only when the tDCS device was active. Participants were informed of when the stimulation was turned on and were instructed not to fall asleep during the scans.

### Behavioural protocol

**Fig. 1:**
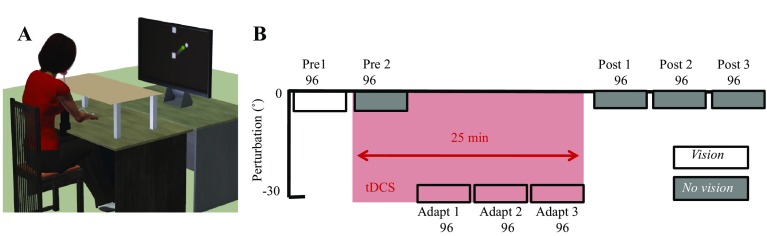
Visuomotor adaptation task. **a** Experimental set up; participants sat behind a table facing a vertically orientated screen placed 105 cm in front of them. **b** Task protocol: Following 2 baseline blocks (each 96 trials: pre 1–2), an abrupt 30° VR was applied to the screen cursor and was maintained across 3 blocks (adapt 1–3). Cerebellar tDCS (anodal/sham) was applied from pre 2 until adapt 3 (pink). Following this, retention was examined by removing visual feedback (grey) for the final 3 blocks (post 1–3)

Participants were seated at a table, with their chin supported by a rest (Fig. 1a), in front of a computer monitor (30-inch; 1280 × 1024 pixel resolution; 105 cm from chin rest). A Polhemus motion tracking sensor (Colchester, VT, USA) was attached to their right index finger and their arm was placed underneath a horizontally suspended wooden board, which prevented direct vision of the arm (Fig. 1a). The visual display consisted of a 1-cm diameter starting box, a green cursor (0.25 cm diameter) representing the position of the subject’s index finger, and a circular white target (0.33 cm diameter). Targets appeared in 1 of 8 positions (45° apart) arrayed radially at 8 cm from the central start position. Targets were selected pseudo-randomly so that every set of eight consecutive trials (one epoch) included all eight target positions. Participants controlled the green cursor on the screen by moving their right index finger across the table top (Fig. 1a). At the beginning of each trial, participants were asked to move their index finger to the start position and a target then appeared. Participants were instructed to make a fast ‘shooting’ movement through the target such that online corrections were effectively prevented. At the moment the cursor passed through the invisible boundary circle (an invisible circle centred on the starting position with an 8 cm radius), the cursor was hidden and the intersection point was marked with a static yellow square to denote the terminal (endpoint) error. In addition, a small square icon at the top of the screen changed colour based on movement speed. If the movement was completed within 100–300 ms, then it remained white. If the movement was slower than 300 ms, then the box turned red (too slow). Importantly, the participants were reminded that spatial accuracy was the main goal of the task. After each trial, subjects moved back to the central start position, with the cursor only reappearing once they were within 2 cm of its location.

#### Visuomotor adaptation

The aim of the behavioural experiment was to replicate the findings of Galea et al. (2011). Therefore, participants were exposed to 8 blocks of 96 trials (12 epochs of all 8 targets). The first 2 blocks acted as baseline and consisted of veridical feedback with (pre1) and without (pre2) online visual feedback (Fig. 1b). During the no visual feedback trials, participants were instructed to continue to strike through the visible target, but received no visual feedback either during or at the end of their movement. Following this, participants were exposed to 3 blocks of trials (adapt 1–3) in which an abrupt 30° counter clockwise (CCW) visual rotation was applied. Finally, to assess retention, three blocks (post-1–3) were performed without visual feedback. tDCS was applied from the start of pre2 and throughout the adaptation blocks, lasting 25 min (Fig. 1b).

### MRS acquisition

**Fig. 2:**
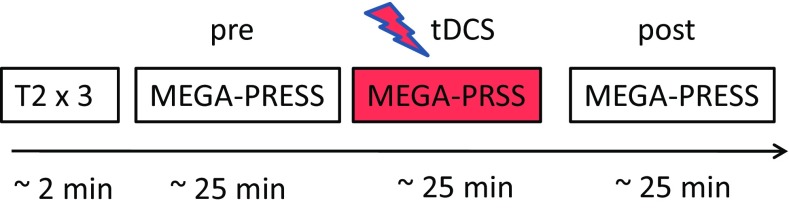
Graphical representation of MRS session using voxel localiser scans (T_2_) and MEGA-PRESS pulse sequence. MRS data was acquired pre-, during, and post-tDCS (lasting 25 min each) performed sequentially within the same individually localised voxel

**Fig. 3:**
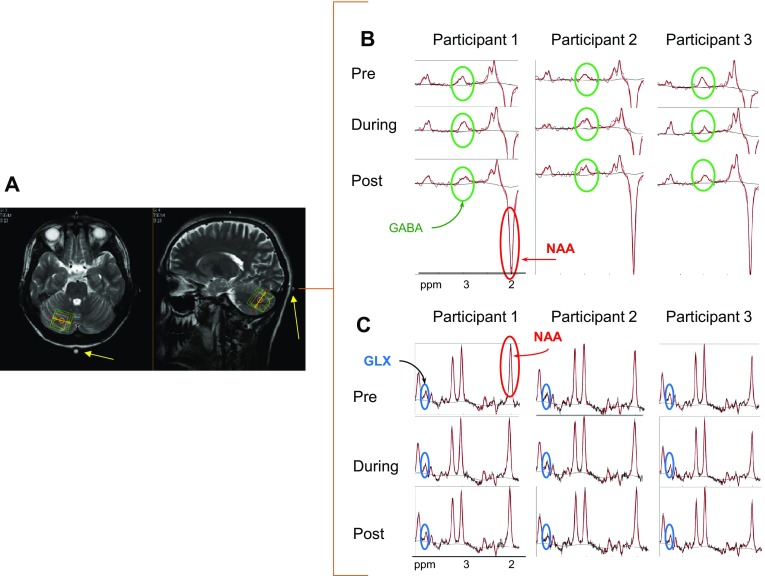
MRS voxel localisation. **a** A single 2 × 2 × 2 cm voxel size was located manually in the posterior part of the right cerebellum underneath the anodal electrode. A cod liver oil capsule (yellow arrow) was situated at the top left edge of the electrode to assist with voxel localisation. Three sets of data were acquired: pre-, during and post- cerebellar tDCS; example MRS spectra are shown for three participants including N-acetylaspartate (NAA) and highlighting the (**b**) GABA and **c** GLX metabolite signals

The anodal group also participated in a MRS session in which data was acquired pre-, during and post-25 min of cerebellar tDCS (Fig. 2) on a Philips Achieva 3T system (Philips Medical Systems, Best, The Netherlands) with a 32-channel radio frequency head receive-coil. The aim of this session was to measure tDCS-induced changes in GABA and Glu concentrations within the cerebellum. Three orthogonal T2-weighted localiser scans (34 slices, 4 mm thickness, and 1 mm gap, voxel size = 0.8 mm × 1.1 mm, 40 s duration) were collected to allow precise manual localisation of the 2 cm × 2 cm × 2 cm MRS single voxel in the posterior part of the cerebellum underneath the electrode. A high-resolution T1-weighted had been acquired in a different session (sagittal, 175 slices, voxel size 1 × 1 × 1 mm, TR/TE = 8.4/3.8 ms, NSA = 1, 10.40 min duration). A cod liver oil capsule was placed on the top right corner of the electrode. As this could be seen in the localizer images, it was used as a marker to aid the placement of the MRS voxel (Fig. 3a).

A GABA signal was measured from the proton spin coherence resonance at 3.0 ppm, accomplished by J-difference editing after scanning using a MEscher–GArwood-Point RESolved Spectroscopy (MEGA-PRESS) (Mescher et al. 1998) sequence with a pulse repetition time (TR) of 2000 ms, echo time (TE) of 68 ms and total duration 25 min. We produced an average GABA spectrum from a total of 512 spectral acquisitions each with a bandwidth of 2150 Hz, sampled at 2048 data points, and with prior water suppression using variable power radio-frequency pulses with optimized relaxation delays (VAPOR) (Tkac et al. 1999) at 4.68 ppm.

To achieve an edited GABA spectral signal without contamination from macromolecules (MM), the two frequency selective 180° RF pulses (Gaussian pulses with duration of 16.5 ms) in the MEGA-PRESS sequence were applied with the centre of the frequency band interleaving between 1.9 ppm (edit-On) and 1.5 ppm (edit-Off) (Henry et al. 2001), across the 512 spectral acquisitions. The edit-Off spectra were subtracted from the edit-On spectra resulting in a spectrum with an unequivocal GABA signal. The acquired edit-Off spectra were also separately analysed to measure concentrations of other metabolites including GLX (Glu + Glutamine (Gln)). Typical spectra identifying GABA and GLX from three participants are shown in Fig. 3b, c. Additional unsuppressed water scans were also acquired to allow corrected metabolite signal quantification. Both metabolites were expressed relative to water concentration.

This study required three separate scans to measure GABA pre-, during- and post- tDCS in a single voxel. To examine the temporal stability and reproducibility of the GABA signal measurements in three subsequent scans, we carried out three test scans on a phantom containing 18 mM of GABA. We found the GABA signal to be highly consistent across the scans. All spectra were aligned and the measured concentration from all three scans were similar: GABA:H_2_O = mean ± standard deviation (stdev) = (1.2 ± 0.11) × 10^−3^. The small stdev confirms the stability of our GABA measurements during in vitro conditions.

### Data analysis

#### Visuomotor adaptation task

Data and statistical analysis was performed using MATLAB (The Math Works, USA) and SPSS (IBM, USA). Index finger position (X & Y position) data was collected at 120 Hz. For each trial, angular hand direction (°) was calculated as the difference between the angular hand position and angular target position at the point when the cursor intersected the 8 cm invisible circle centred on the starting position. During veridical feedback (pre1, Fig. 1b), the goal was for hand direction error to be 0°. However, with the visuomotor transformation (adapt 1–3), hand direction had to compensate; that is, for the − 30° (CCW) visuomotor rotation, a hand direction of + 30° relative to the target was required. Positive values indicate a CW direction, whereas negative values indicate a CCW direction. In addition, reaction time (RT: difference between the target appearing and the participant moving out of the start position) and movement time (MT: difference between reaction time and movement end) were calculated for each trial. We removed any trial in which hand direction, RT or MT exceeded 2.5 standard deviations above the group mean. This accounted for 1.2% of trials. Epochs were created by binning 8 consecutive movements, 1 towards each target.

The angular hand direction (°) of anodal and sham groups was compared for each block of baseline using separate 2-tailed independent *t* tests. For adaptation and retention, separate repeated-measures ANOVAs compared groups (anodal/sham) across blocks (3). Finally, for reaction time (RT) and movement time, two separate repeated-measures ANOVAs compared groups (anodal/sham) across all 8 blocks (Pre 1–2, Adapt 1–3, Post 1–3). The threshold for all statistical comparisons was *P* < 0.05. Effect sizes are reported as partial eta squared for ANOVA and Cohen’s *d* for *t* tests. All data are presented as mean ± standard error of the mean, unless otherwise specified.

#### MRS analysis

Spectroscopy data was analysed using TARQUIN version 4.3.4 (Wilson et al. 2011). First, pre-processing was carried out including inspection and removal of corrupted spectra arising from motion or technical problems. Then, raw data were Fourier-transformed to a spectrum of 2048 data points, the signal was smoothed by a 3 Hz Lorentzian filter, phased and referenced to water signal at 4.7 ppm. Random drift due to scanner instability or subject motion was corrected by aligning the water peak before fitting a Lorentzian–Gaussian (Voigt) line shape model. The amount of drift was plotted and used to assess the quality of acquisition. Scans with less than 10 Hz drift were taken to have acceptable spectra. However, high drift was not the only criterion used to remove data; quality control was performed based on a flat baseline, the shape of the GABA peak in the average spectrum and the smoothness of the residual between the actual data and the fitted model. Signal to noise ratio (SNR) or Cramér–Rao bound (CRLB) were not recommended to be used as quality control in TARQUIN due to the small GABA signal SNR (according to TARQUIN forum discussions). As a result, four subjects were removed from analysis due to an unreliable spectrum and/or poor fitting in one of the three acquisitions (pre-, during, or post-tDCS).

A basis set predefined in TARQUIN was initially constructed based on known peak positions (Voigt function). This basis set was fit to the average spectrum allowing peak amplitudes, widths, and frequencies to be optimized (Wilson et al. 2011). The basis set was then updated with the newly determined frequencies and peak widths and this process of basis set refinement was repeated until fitting resulted in negligible adjustment to the basis set. To detect GABA, all edit-On and edit-Off spectra were averaged separately and then subtracted from each other, but GLX (Glu + Gln) was measured from the average of edit off spectra and Glu extracted from GLX using the predefined basis set in TARQUIN. The reason for using edit off is to avoid subtraction artefacts from the misalignment of the edit-on and edit-off spectra (Evans et al. 2013).

Next, the T1-image of each participant was co-registered to their T2-image using Statistical Parametric Mapping (SPM12) (Friston et al. 1989) and the quality of registration was checked by plotting joint histograms of co-registered T1 vs. T2 images, and by inspection of land marks (specifically on the cerebellum). Then segmentation of the T1 image was carried out using the FMRIB automated segmentation tool (FAST) (Zhang et al. 2001) to calculate the relative volume of each tissue type; grey matter, white matter (WM) and cerebral spinal fluid (CSF) within the MRS voxel. The amplitude of GABA and Glu were corrected for the proportion of GM volume in the voxel by multiplying by $\frac{{{\text{GM}}}}{{{\text{~GM}}+{\text{WM}}+{\text{CSF}}}}$ (Stagg et al. 2011; Kim et al. 2014). Finally, the percentage change ratios for both metabolites for pre- versus during-tDCS, and pre- versus post-tDCS scans were calculated by (100 × (during-pre)/pre) and (100 × (post-pre)/pre) respectively (Stagg et al. 2011).

To assess the modulation of metabolites in response to cerebellar tDCS, repeated-measures ANOVAs compared concentrations of each metabolite pre-, during, and post- tDCS. A Bonferroni correction for multiple comparisons meant the threshold for statistical comparisons was set at *P* < 0.016. All data are presented as mean ± standard error of the mean, unless otherwise specified.

Finally, we examined whether changes in GABA and Glu could predict visuomotor performance. Therefore, partial correlations were carried out between: (1) the change in GABA:H_2_O ratio during tDCS with both total adaptation and retention; (2) the change in GABA:H_2_O ratio change post-tDCS and retention. In both cases, we controlled for Glu:H2O ratio change because Glu is precursor for GABA synthesis. Correlations were also carried out between (3) the change in Glu:H_2_O ratio during tDCS and total adaptation and retention, and (4) the change in Glu:H_2_O ratio change post-tDCS and retention. A Bonferroni correction for multiple comparisons meant the threshold for statistical comparisons was set at P < 0.008.

## Results

### Visuomotor adaptation

**Fig. 4:**
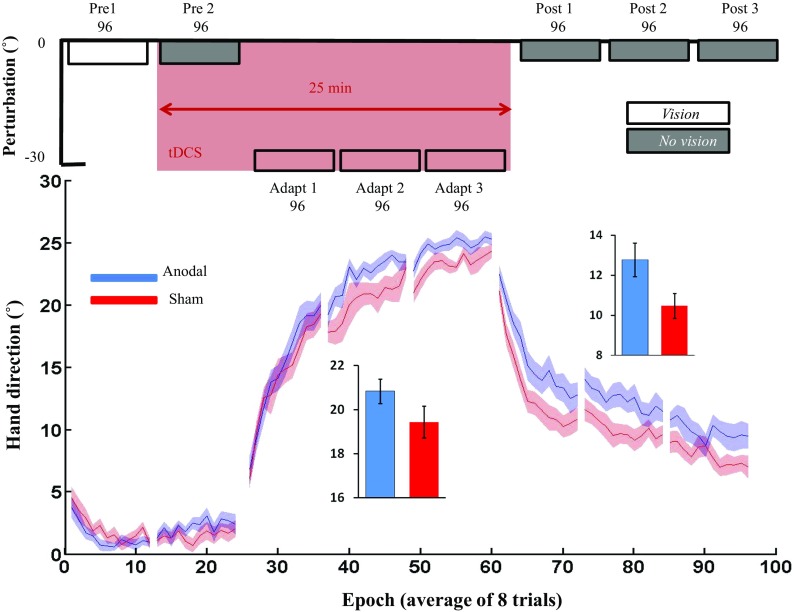
Influence of cerebellar tDCS on visuomotor adaptation. Epoch data (average across 8 trials) for angular hand direction (˚) for the anodal (blue) and sham cerebellar tDCS groups. Positive values indicate CW hand direction. The inset bar graphs indicate mean hand direction for the anodal and sham groups during adaptation (adapt 1–3) and retention (post-1–3). Solid lines, mean; shaded areas/error bars, S.E.M

The performance of 17 anodal and 17 sham participants were compared across all blocks. Both groups behaved similarly during baseline with no significant differences in hand direction between groups during either pre1 (anodal: 1.20 ± 0.22, sham 1.83 ± 0.32; *t*_(32)_ = − 1.4, *p* = 0.1, *d* = 0.08; Fig. 4) or pre2 (anodal: 2.24 ± 0.33, sham: 1.53 ± 0.34; *t*_(32)_ = 0.9, *p* = 0.4, *d* = 0.2). For adaptation, we found no significant differences between the anodal and sham groups. Specifically, there was a significant main effect for blocks (*F*(2,32) = 205.6, *p* < 0.005, *ɳ*^2^ = 0.86), but no significant main effect for group (*F*_(1, 32)_ = 2.3, *p* = 0.14, *ɳ*^2^ = 0.07) or block–group interaction (*F*_(1,32)_ = 0.63, *p* = 0.43, *ɳ*^2^ = 0.02; Fig. 4). Based on these results (total adaptation: anodal = 20.84 SD = 2.3, sham = 19.44 SD = 2.98), a power analysis revealed (*d* = 0.53, power = 0.8) that group sizes of 45 participants would be required to observe a significant result. For retention, we found an unexpected difference between groups whereby the anodal group retained significantly more than the sham group. Specifically, there was a significant main effect for blocks (*F*_(2,32)_ = 114.9, *p* < 0.005, *ɳ*^2^ = 0.78) and group (*F*_(1,32)_ = 4.7, *p* = 0.037, *ɳ*^2^ = 0.13), but no significant block–group interaction (*F*_(1,32)_ = 0.6, *p* = 0.44, *ɳ*^2^ = 0.02; Fig. 4). For RT, there were no significant main effect for group (anodal: 0.43 ± 0.04, sham: 0.39 ± 0.05; *F*_(1,32)_ = 2.02, *p* = 0.2, *ɳ*^2^ = 0.06), blocks (*F*_(2,32)_ = 2.5, *p* = 0.1, *ɳ*^2^ = 0.07), or block–group interaction (*F*_(1,32)_ = 1.2, *p* = 0.3, *ɳ*^2^ = 0.04). Similarly, for MT there were no significant main effect for group (anodal: 0.22 ± 0.08, sham: 0.24 ± 0.08, *F*_(1,32)_ = 3.3, *p* = 0.08, *ɳ*^2^ = 0.09) or block–group interaction (*F*_(1,32)_ = 0.4, *p* = 0.8, *ɳ*^2^ = 0.01), but a significant main effect for blocks (*F*_(2,32)_ = 9.9, *p* < 0.005, *ɳ*^2^= 0.24).

### MRS

#### tDCS did not consistently modulate GABA or glu

**Fig. 5:**
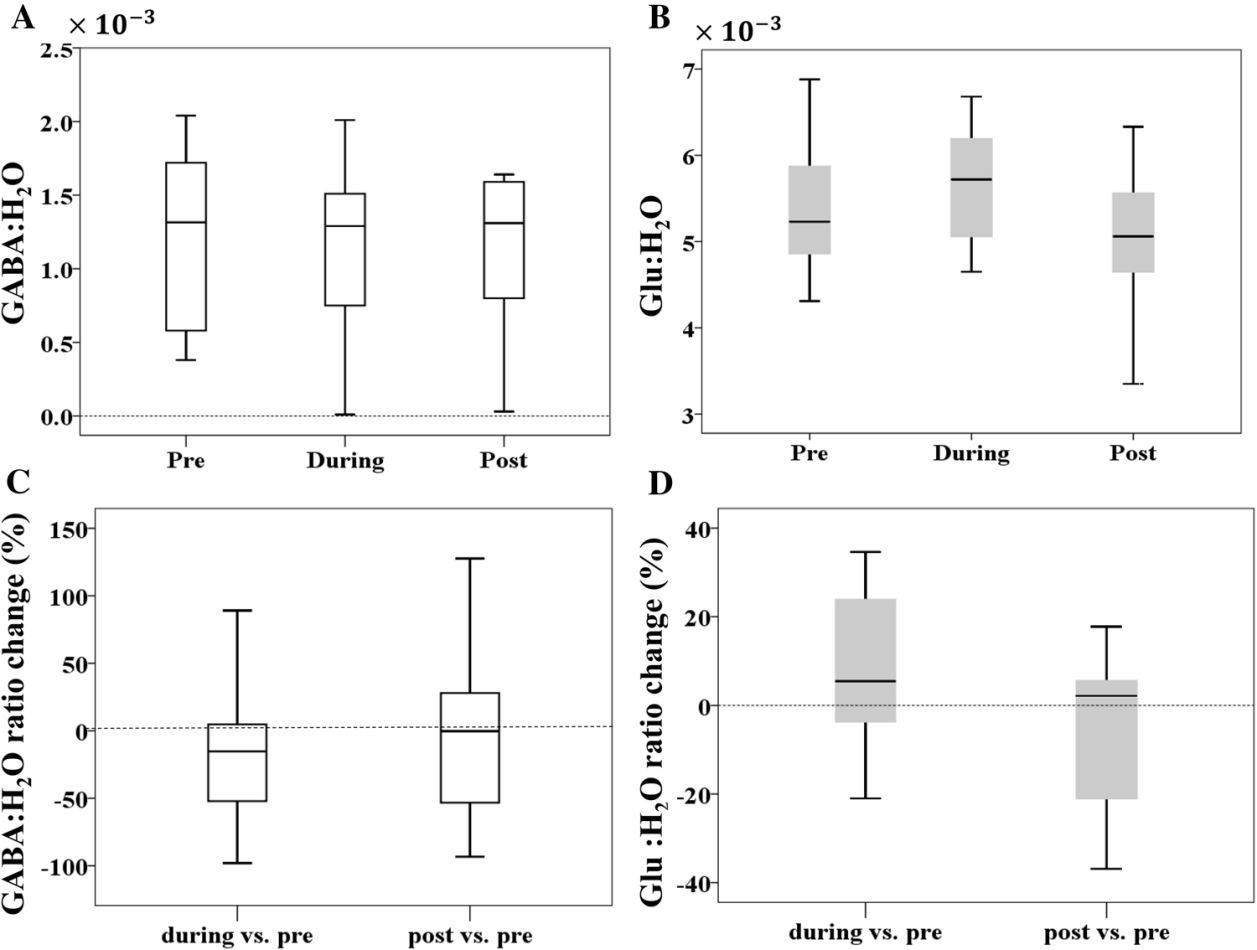
Cerebellar tDCS dependent changes in GABA and Glu. The average **a** GABA:H_2_O and **b** Glu:H_2_O ratio pre-, during and post-cerebellar tDCS. Change (%) in **c** GABA:H_2_O; **d** Glu:H_2_O during and post-cerebellar tDCS relative to baseline (pre-tDCS). The box-plot limits represent the 25th and 75th data percentiles and the middle line represents the median. The error bars represent the range of data

In the anodal group, we measured metabolites within the right posterior cerebellar cortex underneath the anodal electrode at three time-points: pre-, during and post-25 min of anodal cerebellar tDCS. First, grey matter tissue fraction was not significantly different across the three time-points (*F*_(2,24)_ = 0.95, *p* = 0.4, *ɳ*^2^ = 0.07). Crucially, there was no significant change in either GABA:H20 (*F*_(2,24)_ = 0.56, *p* = 0.58, *ɳ*^2^ = 0.04; Bonferroni-corrected threshold *p* = 0.016; Fig. 5a) or Glu:H_2_O (*F*_(2,24)_ = 4.2, *p* = 0.02, *ɳ*^2^ = 0.26; Fig. 5b) across the three time points.

#### tDCS-induced changes in Glu:H_2_O during tDCS were inversely correlated with retention

**Fig. 6:**
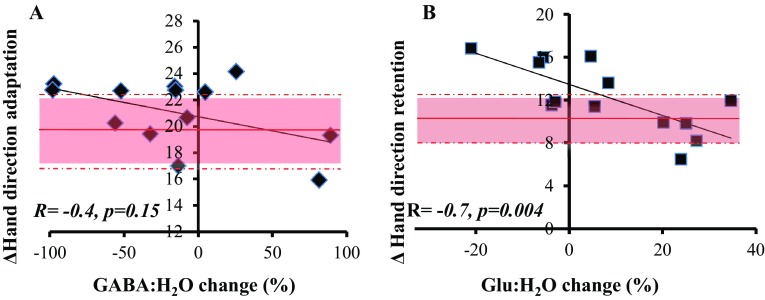
Correlations between MRS and visuomotor adaptation. **a** There was no significant correlation between the changes in the GABA:H20 ratio during cerebellar tDCS and total adaptation. The red line represents the sham group’s mean performance during total adaptation (shaded area = SD across group). **b** A significant negative correlation was observed between changes in the Glu:H2O ratio during cerebellar tDCS and total retention. The red line represents the sham group’s mean performance during total retention (shaded area = SD across group)

Given the large between-subject variability (e.g. from ~ 90% increase to a 100% decrease for GABA:H_2_O and from ~ 20% decrease to ~ 40% increase for Glu:H_2_O during vs. pre; Fig. 5c, d), we went on to examine whether changes in GABA and Glu could predict visuomotor adaptation performance across participants. There was no significant correlation between the change in the GABA:H_2_O ratio during tDCS with total adaptation (*r* = − 0.40, *p* = 0.15; Bonferroni-corrected threshold *p* = 0.008, Fig. 6a) or total retention (*r* = − 0.19, *p* = 0.49), nor between the change in the GABA:H_2_O ratio post-tDCS with total retention (*r* = − 0.07, *p* = 0.81). In addition, there was no significant correlation between the change in the Glu:H_2_O ratio during tDCS with total adaptation (*r* = − 0.08, *p* = 0.78), but there was a significant correlation with total retention (*r* = − 0.74, *p* = 0.004, Fig. 6b). There was also no significant correlation between the change in the Glu:H_2_O ratio post-tDCS and total retention (*r* = − 0.29, *p* = 0.32).

Finally, in a purely explorative nature we observed that cerebellar tDCS led to enhanced performance during the late phase (adapt 3) of adaptation. As previous work has suggested that this part of adaptation is more cerebellar-dependent (McDougle et al. 2015), we asked whether this performance was correlated with changes in either the GABA:H_2_O or Glu:H_2_O ratio during tDCS. There was a significant negative correlation between the change in GABA:H_2_O ratio during tDCS and late (adapt 3) adaptation (*r* = − 0.66, *p* = 0.014), but not in Glu:H_2_0 (*r* = 0.20, *p* = 0.50). Although exploratory, this provides subtle evidence that participants who showed a decrease in GABA during cerebellar tDCS also displayed greater late adaptation.

### Self-reported ratings of attention, fatigue, and sleep

There were no significant differences between groups for the self-reported ratings of attention, fatigue and quality of sleep (Table 1).

**Table 1:** Self-reported rate of attention, fatigue, quality of sleep (1 is poorest and 7 is the maximal), perceived tDCS as active (1) or sham (0) and sleep hours. All the values are averaged and compared using independent *t* tests between the groups, and presented as mean ± standard deviation (SD)

Visuomotor task	Attention	Fatigue	Sleeping hours	Quality of sleep	Active or sham
Anodal	5.3 ± 1.1	3.7 ± 1.5	7.2 ± 1.2	5.1 ± 1.4	0.8 ± 0.3
Sham	4.6 ± 1.1	3.7 ± 1.5	7.2 ± 1.6	4.7 ± 1.7	0.6 ± 0.5
*T* test	*t*_(27)_ = 1.6, *p* = 0.1	*t*_(27)_ = 0.03, *p* = 0.9	*t*_(27)_ = 0.04, *p* = 0.9	*t*_(27)_ = 0.6, *p* = 0.5	*t*_(27)_ = 1.4, *p* = 0.2

## Discussion

This study revealed no statistically significant behavioural differences between anodal and sham cerebellar tDCS groups during visuomotor adaptation, and no consistent change in GABA and Glu in response to concurrent cerebellar tDCS. However, surprisingly, we found cerebellar tDCS led to an improvement in motor memory retention which was strongly correlated with a decrease in Glu during tDCS.

### Cerebellar tDCS did not significantly improve visuomotor adaptation, but enhanced retention

Although participants showed a clear ability to adapt to the novel visuomotor rotation, the expected significant enhancement of adaptation by anodal cerebellar tDCS, that had been shown in various studies (Galea et al. 2011; Hardwick and Celnik 2014; Block and Celnik 2013; Leow et al. 2017), was not observed here. Despite our sample size being in the same range of previously published tDCS papers, a recent study indicates this could be significantly under powered (Minarik et al. 2016). Minarik et al. (2016) showed that with a suggested tDCS effect size of 0.45, the likelihood of observing a significant result with 14 participants per group was approximately 20%. In fact, a power analysis based on our results revealed that we achieved an effect size of 0.53, suggesting group sizes of 45 participants would have been required to observe a significant difference between the anodal and sham tDCS groups. In accordance with this, some previous work indicates that there is substantial variation in the behavioural effect of cerebellar tDCS across participants (Jalali et al. 2017).

Unexpectedly, the anodal group showed greater motor memory retention in comparison to sham tDCS. Although in force-field adaptation it has been shown that cerebellar tDCS influences both the formation of motor memory and its retention (Herzfeld et al. 2014), this effect of stimulation has not been previously shown in similar visuomotor adaptation tasks (Galea et al. 2011; Jalali et al. 2017). At present, we have no clear reason why we observed a positive effect of cerebellar tDCS on memory retention during a visuomotor adaptation task; however, we return to this question when discussing the strong correlation observed between retention and Glu across participants.

### No significant detectable change in GABA or glu in response to cerebellar tDCS

Similar to the behavioural results, there was no consistent group effect of tDCS on GABA or Glu measured within the cerebellum either during or after stimulation. This is in contrast to several previous studies that have shown a significant decrease in GABA in response to M1 tDCS (Stagg 2014, Stagg et al. 2014; Stagg et al. 2011; Kim et al. 2014; Bachtiar et al. 2015), but similar to reports of no significant changes in Glu being observed following M1 tDCS (Stagg et al. 2011; Kim et al. 2014). However, as this is a different brain region with different stimulation duration/intensity it is very difficult to make comparisons. It is possible that cerebellar tDCS simply does not cause consistent between-subject changes in GABA and Glu as it is also shown for M1 tDCS (Tremblay et al. 2016). Alternatively, as each MRS measurement represented the average of 25 min of acquisition we may have been unable to capture any fast or short-lasting changes in these metabolites.

### No correlation between changes in GABA and adaptation, but online cerebellar tDCS reductions in Glu were correlated with motor retention

Although there were no consistent metabolite concentration changes during or post- cerebellar tDCS, we observed large inter-subject variability. Therefore, we examined whether changes in GABA and Glu could predict visuomotor performance with cerebellar tDCS. Our findings demonstrated no significant correlation across participants between changes in GABA during stimulation and total adaptation, however there was an, exploratory, significant negative correlation with the late phase of adaptation. This latter result might confirm the finding by McDougle et al. 2015, who reported that the late phase of adaptation is more cerebellar-dependant (McDougle et al. 2015). As this correlation was specific to GABA, and not Glu, it might suggest a role for GABA in the online effects of cerebellar tDCS during visuomotor adaptation, however, further investigation is required. Specifically, during the current task it is likely that adaptation involved cerebellar-dependent sensorimotor recalibration but also the use of explicit strategies (Taylor et al. 2014). Importantly, it has recently been shown that cerebellar tDCS increases implicit learning only when strategic re-aiming is suppressed during adaptation (Leow et al. 2017). Therefore, it is possible that a stronger relationship between cerebellar tDCS changes in GABA and visuomotor adaptation performance would be observed when using a task that minimised the use of strategies.

Surprisingly, we also found that participants who showed decreases in Glu during cerebellar tDCS within the MRS session showed greater levels of motor memory retention post-tDCS during the behavioural session. At present, it is difficult to explain this correlation. One possibility is that a decrease in Glu reflects a decrease in glutamatergic input into the cerebellar cortex (from mossy fibres and/or granule cells) and would, therefore, lead to reduced activity of Purkinje cells. This would reduce cerebellar brain inhibition (CBI) and enhance M1 function. It is known that excitation of M1 facilitates retention, potentially retaining or consolidating what has been learnt by the cerebellum (Galea et al. 2011; Sami et al. 2014). Although extremely interesting, as the positive effect of cerebellar tDCS on retention was unexpected and contrary to previous literature, we believe it is crucial that future work attempts to replicate the negative correlation between levels of Glu and motor retention.

A major limitation of this study was the lack of a sham tDCS MRS session. As we compared changes in GABA and Glu across MRS scans, it is possible that inter-individual variability in these measures simply reflected either unreliable GABA quantification or natural variations in neurotransmitter levels at rest. Although our small phantom study suggested that we could measure GABA across three scans with little variability, this does not mean that our in vivo measurements of GABA and Glu did not suffer from inter-scan variability. Therefore, to confirm the significant correlation between the changes in Glu and memory retention, future work should include a sham tDCS condition which would enable tDCS-dependent changes in MRS signal to be dissociated from natural changes occurring at rest.

Lastly, not having any measures of participant alertness during data acquisition leaves the possibility that some of the heterogeneity in the observed MRS results could have been driven by variability in the alertness of the participants while lying in the scanner even though according to their report, none of the participants fell asleep. MRS data from a sham tDCS condition would have been useful in assessing this possibility- revealing the GABA and Glu changes which might simply be associated with lying in the scanner for this kind of duration.

## Conclusion

In summary, we found no statistically significant behavioural differences between anodal and sham cerebellar tDCS groups during visuomotor adaptation, and no consistent change in GABA and Glu in response to concurrent cerebellar tDCS. However, cerebellar tDCS did lead to an improvement in motor memory retention which was strongly correlated with a decrease in Glu during tDCS. Thus, this work provides novel insights regarding the neural mechanisms that could underlie cerebellar tDCS. Although interesting, these effects are incompatible with previous literature highlighting the need for replication and limitations in the ability to produce robust cerebellar tDCS effects across participants and between studies.
